# Carbocations and the Complex Flavor and Bouquet of Wine: Mechanistic Aspects of Terpene Biosynthesis in Wine Grapes

**DOI:** 10.3390/molecules200610781

**Published:** 2015-06-11

**Authors:** Henry B. Wedler, Ryan P. Pemberton, Dean J. Tantillo

**Affiliations:** Department of Chemistry, University of California, Davis, One Shields Avenue, Davis, CA 95616, USA; E-Mails: hbwedler@ucdavis.edu (H.B.W.); pemberton@ucdavis.edu (R.P.P.)

**Keywords:** wine, terpene, biosynthesis, quantum chemistry, mechanism, aroma

## Abstract

Computational chemistry approaches for studying the formation of terpenes/terpenoids in wines are presented, using five particular terpenes/terpenoids (1,8-cineole, α-ylangene, botrydial, rotundone, and the wine lactone), volatile compounds (or their precursors) found in wine and/or wine grapes, as representative examples. Through these examples, we show how modern computational quantum chemistry can be employed as an effective tool for assessing the validity of proposed mechanisms for terpene/terpenoid formation.

## 1. Introduction

**Figure 1:**
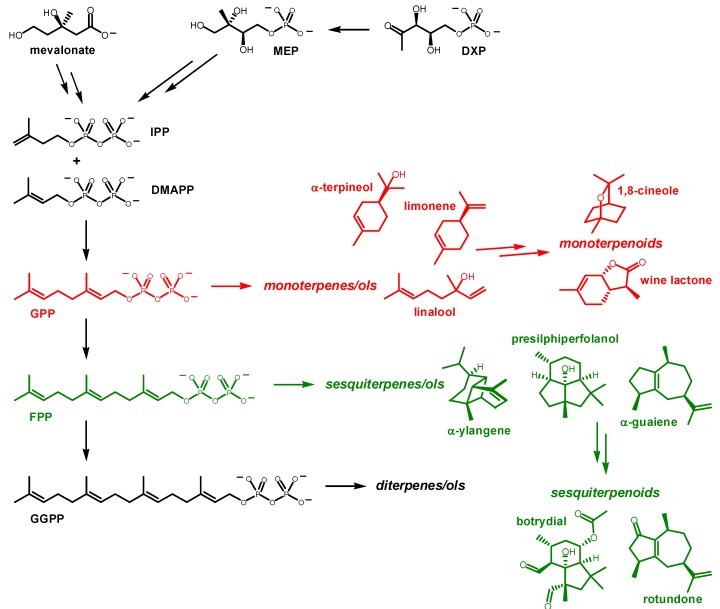
Overview of chemical transformations in terpene biosynthesis.

Terpenes are hydrocarbon natural products constructed from connected isoprene units (each containing five carbons). They are often polycyclic and are derived from rearrangement, involving carbocation intermediates, of acyclic precursors by terpene synthase/cyclase enzymes [1,2,3,4,5]. For example, monoterpenes (10 carbons) are synthesized from geranyl diphosphate (**GPP**), while sesquiterpenes (15 carbons) are produced from farnesyl diphosphate (**FPP**) and diterpenes (20 carbons) from geranylgeranyl diphosphate (**GGPP**; Figure 1). Red and white wines predominantly contain monoterpenes and sesquiterpenes. Terpenes are biosynthesized in the grapevine primarily in flowers and berries. Terpenes produced in grape berries and flowers are thought to play roles in defense against pathogens, attraction of seed dispersing animals, and attraction of pollinators [6,7,8]. These terpenes also create complex flavor and aroma in finished wines. Two pathways can generate **GPP**, **FPP**, and **GGPP**: (1) the mevalonate (MVA) pathway and (2) the 2-*C*-methyl-D-erythritol 4-phosphate/1-deoxy-D-xylulose 5-phosphate (MEP/DXP) pathway (Figure 1) [9,10,11,12]. Each pathway employs a unique means of generating isopentenyl pyrophosphate (**IPP**) and dimethylallyl diphosphate (**DMAPP**), which are subsequently combined to form **GPP** by geranyl diphosphate synthase. **GPP** itself is a substrate for farnesyl diphosphate synthase. In wine grapes, *Vitis vinifera*, **GPP** and **FPP** are biosynthesized via the MEP/DOXP pathway in the mitochondria and the MVA pathway in the cytosol, respectively [13].

Removal of the pyrophosphate group from **GPP** or **FPP** by terpene synthase/cyclase enzymes in *vinifera* grape vines generates carbocationic intermediates that rearrange within the enzyme active sites to form monoterpenes and sesquiterpenes [1,2,3,4,5]. Most compounds derived from terpene biosynthesis in wine grapes are actually terpenoids, which are terpenes or terpene alcohols (terpene synthases can produce alkenes via deprotonation or alcohols via water capture) that have undergone chemical transformation(s), typically oxidation (sometimes promoted by enzymes, sometimes not). Genomes for the various *Vitis vinifera* grape varietals have been mapped, with an emphasis on identifying terpene synthase/cyclase coding regions and sub-families [8,14,15].

**Scheme 1:**
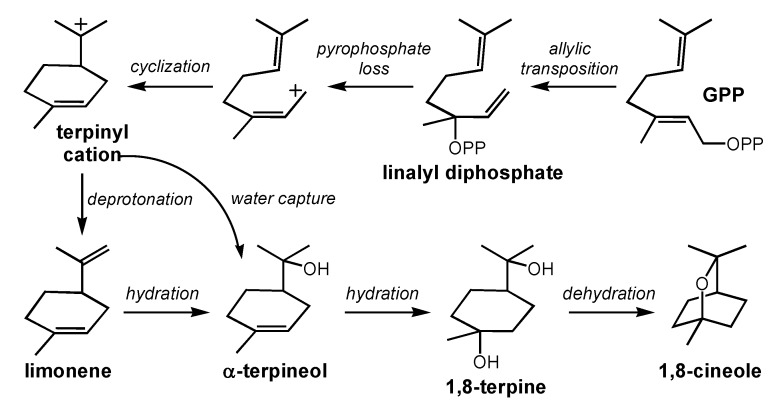
1,8-Cineole-forming carbocation cascade.

As described by Marais, both red and white wines contain numerous terpenes that contribute most of the aroma and flavor in wine [16], although non-terpene compounds, commonly esters and mercaptans, also contribute [17]. Herein we will describe typical chemical mechanisms at play in the biosynthesis of terpenes and terpenoids in wine grapes. The representative compounds we will discuss are: (1) **1,8-Cineole**. The characteristic eucalyptus aroma in Australian Syrah and other full-bodied red wines comes from the monoterpenoid 1,8-cineole, also known as eucalyptol (Figure 1) [18,19,20]; (2) **α-Ylangene**. α-Ylangene (Figure 1) is a sesquiterpene found in various red and white grapes, and is likely the precursor to other terpenoid products found in wine [21]; (3) **Botrydial**. *B. cinerea* is a fungal parasite that can destroy entire vineyards. Botrydial, derived from the sesquiterpene alcohol presilphiperfolanol (Figure 1), is responsible for the phytopathogenetic development of *Botrytis cinerea* and is found on the skins of *Botrytis* infected grape clusters [22,23,24,25]; (4) **Rotundone**. A ubiquitous terpenoid in red wine varietals, rotundone is a derivative of the sesquiterpene α-guaiene (Scheme 1). Rotundone is found in Syrah, Cabernet Sauvignon, Zinfandel, Merlot, and other red *vinifera* varietals (Figure 1) [26,27,28,29,30]. Oxidation of α-guaiene produces rotundone in red wines that are allowed to oxidize [28]. The black pepper aroma in red wines that are allowed ample breathing time prior to consumption has been shown to result from rotundone; (5) **Wine lactone**. Many monoterpenes and monoterpenoids, including linalool, provide crisp, floral aromas to wines from cool climates [31,32]. The wine lactone (Figure 1) is a monoterpenoid lactone that has been isolated from many northern European white and red wine varietals [33]. Experiments have shown that it is also prevalent in numerous other foods including clementine peel, pears, apples, black pepper, and many citrus fruits [33,34].

For some of the compounds mentioned above (**1**–**3**), quantum chemical calculations have been used to tease out subtle features of the chemical mechanisms leading to their hydrocarbon skeletons [1,35,36,37]. These studies will be highlighted below, in hopes of showing the power of computational chemistry techniques in addressing mechanistic problems in biosynthesis. The other compounds discussed (**4**–**5**) would be good targets for future study using computational quantum chemistry. The computational approach generally employed involves mapping out reaction coordinates for terpene/terpenoid-forming reactions by optimizing the geometries, and calculating the relative energies, of potential minima (intermediates) and transition state structures involved in formation of terpene skeletons. These structures are then connected with intrinsic reaction coordinates (IRCs; steepest descent pathways from transition state structures to connected minima); in doing so, barriers for each chemical step are predicted and can be assessed as to their viability at biologically relevant temperatures. In some cases, dynamic effects on reactivity are also treated [38,39,40,41,42]. In general, these rearrangements are studied in the gas phase, *i.e.*, the intrinsic, enzyme-free reactivity is elucidated. While this is a huge approximation, studies have shown that it is often a reasonable one, especially for carbocation cyclizations/rearrangements; e.g., mechanistic predictions made on the basis of inherent reactivity have been validated experimentally [43]. The success of this approximation is likely due to both the (generally) nonpolar nature of the carbocation-binding regions of terpene synthase active sites [42,44], and the low barriers (*i.e.*, fast rates) for carbocation reactions [1,35,36,37]. In general, barriers of <15 kcal/mol, often much lower, are predicted for carbocation rearrangement events: hydride shifts, alkyl shifts, carbocation/alkene cyclizations, and intramolecular proton transfers [39]. These events can occur simultaneously (via concerted and synchronous processes) or one after the other (via stepwise or concerted but asynchronous processes) [1,35,36,37]. While terpene synthase enzymes appear not to be necessary, in general, for facilitating carbocation rearrangement steps, they are necessary for generating the initial carbocation for a given rearrangement, preorganizing the conformation of this carbocation and preventing premature quenching of carbocations by deprotonation or trapping by nucleophiles [42].

## 2. Representative Examples

### 2.1. 1,8-Cineole

1,8-Cineole (Figure 1), also known as, eucalyptol, is responsible for the eucalyptus and minty aromas in red wine [18,45]. 1,8-Cineole is most common in Australian wines due to the high volume of eucalyptus trees surrounding vineyard sites [19]. Capone *et al*. explained that high concentrations of airborne 1,8-cineole enter the vine and add to the ripe fruit, leaves, and stems, resulting in higher 1,8-cineole concentrations in the finished wine. It has long been debated whether the eucalyptus aroma of 1,8-cineole is a positive attribute, or should be considered a taint in red wine. Consumer rejection thresholds were analyzed using a sensory panel and wine samples spiked with increasing concentrations of 1,8-cineole [46]. Results estimate that the consumer rejection threshold of 1,8-cineole is near 27.2 ppb. Though most 1,8-cineole enters grapes via external sources, it can potentially be biosynthesized by the grape itself [47]. 1,8-Cineole is a derivative of limonene (Figure 1), whose formation mechanism has been studied with a variety of quantum chemical methods [42,43]. After enzyme-promoted isomerization of **GPP** to linanlyl diphosphate and loss of pyrophosphate, cation-alkene cyclization forms the terpinyl cation (Scheme 1); this cyclization is predicted to be exothermic by greater than 13 kcal/mol. The route from the terpinyl cation to 1,8-cineole is not entirely clear, however, and may differ in different biological environs. Deprotonation of this cation produces limonene, which has been shown to be converted, under acidic conditions, to 1,8-cineole via hydration of both of its C=C double bonds (to form a compound called 1,8-terpine) and cyclodehydration [48]. Formation of 1,8-terpine is thought to involve α-terpineol as an intermediate. These reactions appear to be reversible in wine. Alternatively, the terpinyl cation could be captured directly by water in the enzyme active site to produce α-terpineol [48].

### 2.2. α-Ylangene

**Scheme 2:**
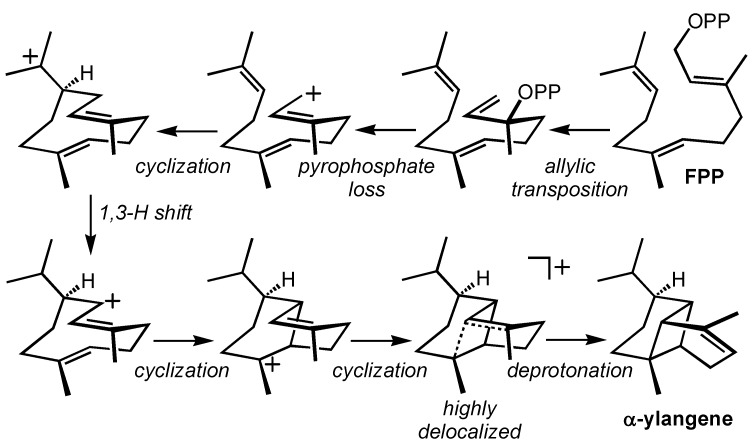
α-Ylangene-forming carbocation cascade.

α-Ylangene (Figure 1) has been identified as a marker for peppery aroma in Shiraz grapes, despite apparently not contributing to this aroma itself, and has been found in other grape varieties [21,49]. The carbocation cyclization/rearrangement cascade that leads to α-ylangene (Scheme 2) is representative of the sort of cascades that lead to most terpenes. The mechanism of this transformation was studied using density functional theory calculations (mPW1PW91/6-31+G(d,p)//B3LYP/6-31+G(d,p)) [37,50]. The results of these calculations indicated that conversion of the macrocyclic cation in Scheme 2 (top left) to α-ylangene via a 1,3-hydride shift → carbocation/alkene cyclization → carbocation/alkene cyclization → deprotonation mechanism is energetically viable; a small overall barrier of approximately 3 kcal/mol and an exothermicity of nearly 30 kcal/mol (to the final carbocation) were computed. After a large drop in energy (approximately 20 kcal/mol) associated with formation of an allylic cation via the 1,3-hydrogen shift, the next cyclization was predicted to have a small barrier (approximately 2 kcal/mol) and be exothemic by several kcal/mol. The final two carbocations were predicted to exist on a flat region of the potential energy surface, with the final carbocation showing delocalization consistent with a hybrid cyclobutylcarbinyl/bishomoallylic cation structure. [50] As is typical for many terpene-forming carbocation reactions, α-ylangene formation is driven forward by the exchange of π-bonds for σ-bonds and increased delocalization of both charge and electron density.

### 2.3. Botrydial

**Scheme 3:**
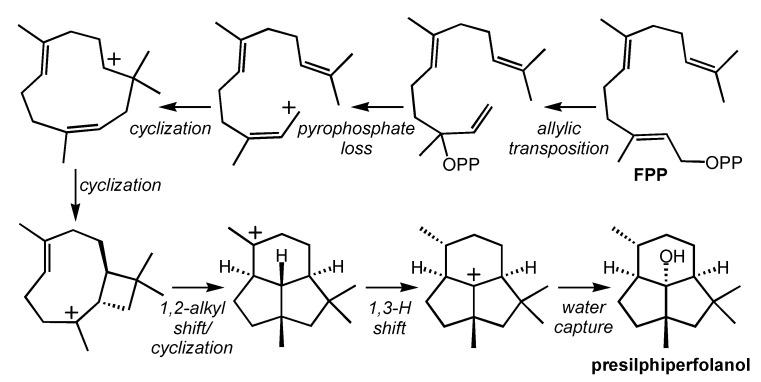
Presilphiperfolanol-forming carbocation cascade.

The toxic sesquiterpenoid botrydial is formed from oxidation of presilphiperfolanol (Figure 1). [22] The presilphiperfolanol-forming carbocation cascade (Scheme 3) was studied in depth using density functional theory calculations (mPW1PW91/6-31+G(d,p)//B3LYP/6-31+G(d,p)) [37,51,52]. On the basis of the results of these calculations it was proposed that the carbocation rearrangement is preceded by rearrangement of the allylic diphosphate of **FPP** (Scheme 3, top right). While this type of allylic transposition is common in terpene biosynthesis (e.g., Scheme 1 and Scheme 2), it was not thought to be necessary for presilphiperfolanol formation. The theoretical work indicated, however, that cyclization to directly from the cyclobutylcarbinyl intermediate (Scheme 3, bottom left) in a conformation productive for subsequent rearrangement required attack by a (*Z*)-alkene. This cyclobutylcarbinyl cation can then rearrange through a concerted process involving the asynchronous combination of an initial ring-expanding 1,2-alkyl shift followed by cyclization; by combining these two events into a concerted process, a previously proposed secondary carbocation intermediate is avoided. Avoidance of secondary carbocations as minima on potential energy surfaces by combining multiple bond-forming/breaking events into concerted processes is a feature shared by many terpene-forming carbocation rearrangements [1,35,36,37]. All carbocation cyclization/rearrangement steps in this mechanism were predicted to be exothermic and have barriers of less than 10 kcal/mol. The final carbocation in this transformation is trapped by water to form a sesquiterpene alcohol.

### 2.4. Rotundone

Rotundone, a guaiene-derived sesquiterpenoid, is responsible for the distinctive peppery aroma in wine and spices. Its odor threshold is extraordinarily low (16 ng/L in wine, 8 ng/L in water) [27]. To put this odor threshold in perspective, 5 mL of rotundone would be enough to make all wine produced annually in Australia taste distinctly of pepper. Rotundone is biosynthesized in the grape berry, and becomes most prominent after véraison [27]. Rotundone concentration is also higher in fruit grown at high elevations with harsh, exposed growing conditions. Many Australian red wines are described as being peppery because of high rotundone concentrations. Rotundone is common in grapes across many regions: other peppery wines include California Zinfandel, many California and French Syrah-Granache blends, Bourdieu varietals, *etc*. Rotundone and the pepper flavor/aroma are only present in red wines. Rotundone has been synthesized by the aerial (*i.e.*, non-enzymatic) oxidation of α-guaiene [28], consistent with the observation that the peppery aroma often increases when wine is left in a glass over time to oxidize.

**Scheme 4:**
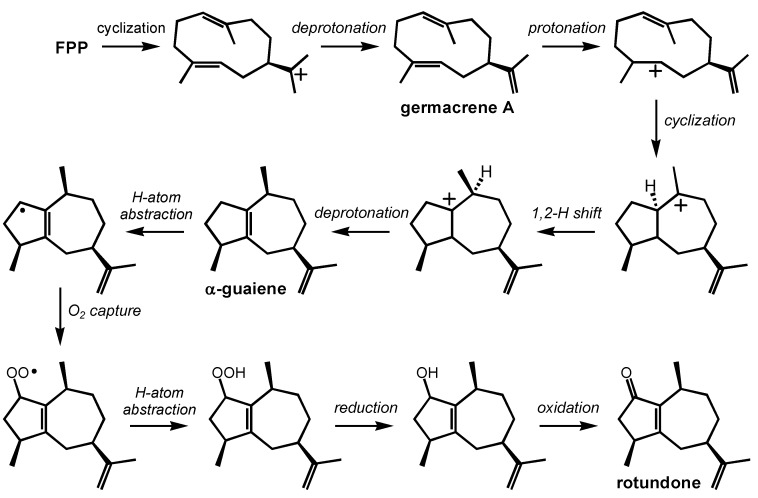
Proposed biosynthetic formation of rotundone: α-guaiene-forming carbocation cascade followed by radical-initiated oxidation.

Several questions about rotundone formation could be addressed using quantum chemical calculations. First, consider the putative carbocation cyclization/rearrangement mechanism shown in Scheme 4. In this mechanism, a deprotonation/reprotonation process, via a germacrene intermediate, is shown, but it is not clear whether this process could be replaced by a concerted intramolecular proton transfer [39]. This proton transfer process—be it concerted or stepwise—would result in a secondary carbocation (Scheme 4, top right). Secondary carbocations are often avoided as intermediates [1,35,36,37], so it is possible that proton transfer is combined with cyclization. Whether or not formation of one particular diastereomer is energetically preferred upon cyclization is also not clear. Quantum chemical calculations of the sort described above could solve these puzzles. Once α-guaiene is formed, non-enzymatic oxidation can convert it to rotundone [28]. A putative mechanism for this process, analogous to a mechanism for lipid peroxidation, is also shown in Scheme 4 (bottom). In this process it is not clear why abstraction of an allylic hydrogen atom to form the specific allylic radical shown predominates; abstraction from three other allylic positions are also possible. Once the allylic radical is formed, it is not clear why O_2_ capture at the specific site shown is preferred, nor is it clear if there is diastereoselectivity for the capture step. Again, quantum chemical calculations could shed light on these issues.

### 2.5. Wine Lactone

**Scheme 5:**
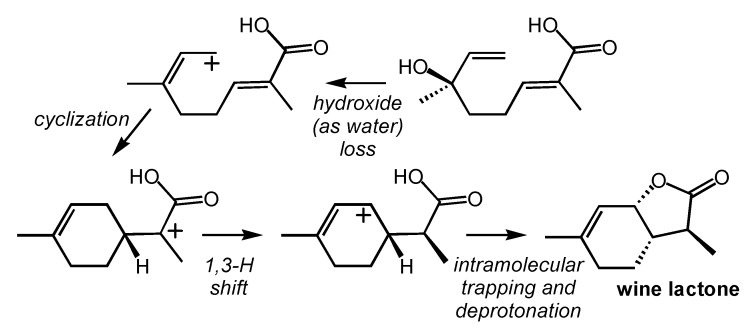
Proposed mechanism for formation of the wine lactone.

Formation of the wine lactone, another potent odor compound [34], was proposed to proceed as shown in Scheme 5 [53]. An oxidized form of linalool (Figure 1) was proposed to lose water to form an allyllic carbocation. Subsequent cyclization to form an α-carboxyl carbocation was proposed to be followed by a 1,3-hydride shift and intramolecular carbocation trapping. Several aspects of this mechanism are unusual. First, it is not clear whether or not loss of allylic delocalization upon cyclization will be fully counterbalanced by exchange of a π-bond for a stronger σ-bond. Second, the stability of the resulting carbocation is not easy to predict, since it is next to a carbonyl and may (as suggested by Wüst and co-workers) engage in nonclassical delocalization [44,50,54,55,56,57,58,59,60,61]. The validity of this proposed mechanism could readily be assessed using computational quantum chemistry. An alternative (perhaps competitve) process would involve oxidation of limonene or α-terpineol rather than linalool (Figure 1); the viability of such a process has not yet been assessed. In addition, mechanisms that make use of α,β-unsaturated carbonyl compounds as Michael acceptors might be involved, and their energetic viability could also be evaluated using quantum chemical computations.

## 3. Conclusions and Outlook

Terpenes and terpenoids often possess unique aromatic and organoleptic properties and they are often the cause of the primary aromas in strongly smelling substances. Most essential oils, for instance, contain terpenes and terpenoids. Wine is a complex blend of natural products including a multitude of terpenes and terpenoids. Both red and white wines contain arrays of terpenes and terpenoids, both on grapes pre-harvest and in finished wines. We have attempted to show herein how modern computational quantum chemistry can be applied to elucidating mechanisms for formation of terpenes and terpenoids present in wine and hope studies along these lines will continue.
